# Injective hydrogel encapsulating dental pulp stem cells for the treatment of traumatic optic nerve injury

**DOI:** 10.3389/fbioe.2025.1528749

**Published:** 2025-02-25

**Authors:** Lihua Luo, Zhenjie Xing, Yao Li, Ben Wang, Na Dong, Jiayi Sun, Shuimiao Wang, Yidi Xu, Yan He, Lingli Li, Kaihui Nan, Qingsong Ye

**Affiliations:** ^1^ School and Hospital of Stomatology, Wenzhou Medical University, Wenzhou, Zhejiang, China; ^2^ The Eye Hospital, School of Ophthalmology and Optometry, Wenzhou Medical University, Wenzhou, Zhejiang, China; ^3^ Tianyou Hospital, Wuhan University of Science and Technology, Wuhan, China; ^4^ Center of Regenerative Medicine, Renmin Hospital of Wuhan University, Wuhan, China; ^5^ Institute of Stem Cells and Tissue Engineering, School of Stomatology, Wenzhou Medical University, Wenzhou, China

**Keywords:** dental pulp stem cells, traumatic optic nerve injury, retinal ganglion cells, basic fibroblast growth factor, hydrogel

## Abstract

**Objectives:**

The study aimed to evaluate the effect of GeLMA/bFGF hydrogel loaded with dental pulp stem cells (DPSCs) on the repair and regeneration of traumatic optic nerve injury.

**Materials and methods:**

GeLMA/bFGF hydrogel was photo-cross-linked by LED light. The physical–chemical properties and cytocompatibility of GeLMA/bFGF hydrogel after being squeezed (GeLMA/bFGF-SQ) were evaluated by SEM and degradation analyses, as well as live/dead and CCK-8 assays, respectively. The axon growth of PC12 cells was evaluated by MAP2 staining. The GeLMA/bFGF/DPSCs-SQ hydrogel was injected *in situ* into the lesion site to observe the repair of optic nerve injury. The number of retinal ganglion cells (RGCs) was calculated by βIII-tubulin staining. The length of regenerated axons was observed by CTB staining. Tissue recovery and axon growth of the optic nerve were observed by HE and TEM analyses, respectively.

**Results:**

GeLMA/bFGF-SQ hydrogel had a porous structure and great degradability, as well as good cytocompatibility. Meanwhile, DPSCs-conditioned medium (DPSCs-CM) could promote the axon growth of PC12 cells. Moreover, the number of RGCs and the regeneration of axons of the optic nerve were the highest in the GeLMA/bFGF/DPSCs-SQ group. HE and TEM data revealed abundant newly and orderly arrangement of optic nerve axons that was observed in the damaged area of the GeLMA/bFGF/DPSCs-SQ group.

**Conclusion:**

Transplantation of GeLMA/bFGF/DPSCs-SQ *in situ* provided an appropriate microenvironment for the repair and regeneration of injured optic nerves. Moreover, DPSCs combined with bFGF protected the RGCs from apoptosis and promoted optic nerve regeneration by secreting a series of neurotrophic factors.

## 1 Introduction

At present, traumatic optic nerve injury is one of the most common neuropathies, which represents a leading cause of permanent blindness in clinic (Chung et al., 2016). Currently, glucocorticoid therapy and surgery are the main strategies for the treatment of optic nerve injury (Habibi et al., 2023; Wladis et al., 2021). However, these methods have many disadvantages, such as drug toxicity, the second trauma, facial bone loss, intraorbital infections, and unpleasant scarring (Chen et al., 2022; Maxwell et al., 2022). Hence, new strategies of repairing and regenerating the injured optic nerve have become hotspots in ophthalmic research.

Retinal ganglion cells (RGCs) are the innermost layer of cells of the neural net, and their axons construct the optic nerve. Studies have demonstrated that the pathological basis of optic nerve injury is the irreversible damage of the optic nerve and the subsequent apoptosis of RGCs (Khan et al., 2021; Huang et al., 2019). Once optic nerve injury occurs, the lack of neurotrophic factors such as ciliary neurotrophic factor (CNTF), nerve growth factor (NGF), brain-derived neurotrophic factor (BDNF), and vascular endothelial growth factor (VEGF) in the surrounding microenvironment will inhibit the nerve growth, eventually causing the death of RGCs (Fudalej et al., 2021). Therefore, the improvement in the surrounding microenvironment is the major prerequisite for the regeneration of optic nerve injury to ensure the survival of RGCs.

Recently, stem cell-based therapy has garnered an increase in attention as a promising approach for the repair of damaged optic nerves (Mead and Tomarev, 2017). Dental pulp stem cells (DPSCs), one of the mesenchymal stem cells (MSCs), have emerged as a notable candidate due to their ease of acquisition, lack of ethical issues, and capacity for rapid replication (Liao et al., 2023). In addition, DPSCs possess the ability to secrete multiple nutrient factors (e.g., NGF, BDNF, and VEGF) to improve the microenvironment in the lesion site (Hata et al., 2020). Meanwhile, the capacity of DPSCs to produce nutrient factors is significantly higher than that of other MSCs such as bone marrow mesenchymal stem cells (BM-MSCs) (Song et al., 2015; Mead et al., 2013). Moreover, DPSCs, which are derived from the cranial neural crest, express lots of neural-related surface markers that may become attractive seeded cells for the treatment of neural diseases (Irfan et al., 2022; Wang et al., 2020). Studies have shown that intravitreal injection of DPSCs can significantly improve the survival of RGCs after optic nerve injury by regulating neurotrophin secretion (Mead et al., 2013).

It is very important to ensure the survival and proliferation of transplanted cells in the lesion site. The fibroblast growth factor (FGF) family, consisting of 22 growth factors, has a wide range of applications in the field of repair and regeneration of tissue damages (Farooq et al., 2021; Mossahebi-Mohammadi et al., 2020). Among them, the basic fibroblast growth factor (bFGF) has received the most attention in the study of promoting axonal regeneration, enhancing angiogenesis, and mobilizing endogenous neural stem cells (Zheng et al., 2021; Zhu et al., 2021). Our previous study demonstrated that the addition of bFGF in the culture medium could reduce the apoptosis of DPSCs (Luo et al., 2021b). Currently, there is a lack of research reporting DPSCs combined with bFGF in the treatment of optic nerve injury.

However, bFGF is one of the protein molecules with some disadvantages, including a short half-life, easy degradation, and limited acting time (Arunkumar et al., 2019; Khalil et al., 2020; Lee et al., 2021). Therefore, an appropriate carrier needs to be used for guaranteeing the survival of DPSCs and the sustained release of bFGF in the injured site of the optic nerve. Hydrogels, with unique three-dimensional networks, are beneficial for the adhesion, growth, and proliferation of transplanted cells (Ma and Huang, 2020). Moreover, hydrogels provide a mild water microenvironment, which is suitable for encapsulating biologically macromolecular drugs to avoid their degradation and inactivation (Shan and Wu, 2024). In our previous study, gelatin methacryloyl (GeLMA) hydrogel, which was prepared by grafting gelatin with methacrylic anhydride, had UV photo-cross-linked property and possessed great cytocompatibility with DPSCs. Meanwhile, 5% GeLMA hydrogel had lower mechanical property and was suitable for injection *in situ* (Luo et al., 2021a). In addition, the cytocompatibility of GeLMA hydrogel via photo-cross linking with a blue-light emitting diode (LED) is obviously better than that of GeLMA hydrogel cross-linked with UV light initiation (He et al., 2024).

Thus, in this study, we fabricated 5% GeLMA hydrogel encapsulating DPSCs and bFGF via cross-linking with LED light initiation and then *in situ* injected it into the injured site for the treatment of optic nerve injury. The purpose of this study was to determine whether the 5% GeLMA hydrogel loaded with bFGF after extrusion could provide a continuously active microenvironment for the survival and proliferation of DPSCs and further played important roles in the repair and regeneration of the optic nerve.

## 2 Materials and methods

### 2.1 Synthesis of gelatin methacrylate (GeLMA)

GeLMA powder was prepared as previously described (Luo et al., 2021a). First, 20 g of gelatin (type A, Sigma-Aldrich, United States) was dissolved in 200 mL phosphate-buffered saline (PBS, Gibco, United States) under magnetic stirring at 50 °C. Then, the gelatin solution was mixed with 2 mL of methacrylic anhydride (MA, Sigma-Aldrich, United States), which was added at a rate of 0.5 mL/min. After 3 h, the suspension was placed in a 1-kDa dialysis tube and dialyzed at 50 °C for 5 days. The final GeLMA macromer was lyophilized and stored at room temperature for further use.

### 2.2 Fabrication of GeLMA/bFGF hydrogels

To fabricate GeLMA/bFGF hydrogels, GeLMA powder was dissolved in deionized water to create a solution with a 5% concentration. Then, lithium phenyl (2,4,6-trimethylbenzoyl) phosphinate (LAP, Tokyo Chemical Industries, Japan), a photo-initiator, was added to this solution at the ratio of 16:1, according to our previous report (He et al., 2024). The mixture was heated for 1 h at 50 °C until the solution was clear. Eventually, the GeLMA solution was poured on a specific silicone mold and exposed to LED light for 2 min to form GeLMA hydrogel. In addition, 20 ng/mL of bFGF was added to this mixed solution before light curing to construct the GeLMA/bFGF hydrogel.

### 2.3 Scanning electron microscopy (SEM)

Morphological properties of these hydrogels were observed by scanning electron microscope (SEM, HITACHI, Japan). The hydrogels were divided into three groups: the GeLMA group as the GeLMA hydrogel received no treatment, GeLMA-SQ group as the GeLMA hydrogel was injected by a 1-mL syringe, and GeLMA/bFGF-SQ group as the GeLMA/bFGF hydrogel was injected by a 1-mL syringe. All samples were flash frozen in liquid nitrogen and lyophilized by a vacuum freeze dryer for 2 days. As for SEM observation, the fracture surface was sputtered with platinum. The images were quantified by ImageJ (Bethesda, United States).

### 2.4 Degradation analysis

The degradation rate of hydrogels was determined by using a weight loss method, as previously described (Liu et al., 2017). In brief, the lyophilized hydrogels (M0) were added to 10 μg/mL of type-I collagenase and kept in the shaker incubator (150 rpm/min) for 2, 6, 12, and 24 h at 37 °C. Then, the hydrogels were lyophilized and weighed at predetermined intervals (M1). The degradation rate was calculated using the following equation:

$$ML\left(\%\right)=\frac{M0-M1}{M0}\times 100\%.$$

### 2.5 DPSC viability and proliferation analyses

DPSCs were obtained and cultured as previously described (Luo et al., 2023). In brief, the pulp tissues were cut into small pieces and digested with the mixture of collagenase type I (Gibco, United States) and dispase (Sigma, Germany). After 1 h, the cell suspensions were cultured with α-modified Eagle’s medium (α-MEM, Gibco, United States) supplemented with 20% fetal bovine serum (FBS, Gibco, United States) and 1% penicillin/streptomycin. The culture medium was changed every 3 days. The viability and proliferation of DPSCs which were co-cultured with hydrogels (GeLMA, GeLMA-SQ, and GeLMA/bFGF-SQ) were investigated by a live/dead viability/cytotoxicity kit (Invitrogen, United States) and a CCK-8 assay kit (Dojindo, Japan), respectively. In brief, DPSCs were dispersed in GeLMA and GeLMA/bFGF solutions and photo-cross-linked to create GeLMA-DPSCs and GeLMA/bFGF-DPSCs hydrogels. The GeLMA-DPSCs hydrogel, which was directly plated into the 96-well plate, was labeled as the GeLMA group. The GeLMA-DPSCs and GeLMA/bFGF-DPSCs hydrogels were injected into the 96-well plate by 1-mL syringes, which were coded as GeLMA-SQ and GeLMA/bFGF-SQ groups, respectively. After 1, 3, and 5 days, 10% CCK-8 solution was added and incubated for another 1 h. An absorbance microplate reader was used to measure the OD values at 450 nm. As for live/dead staining, the hydrogels would be removed and stained according to the manufacturer’s instructions. All samples were examined and captured by a fluorescent microscope (Carl Zeiss, Germany), and ImageJ was used to determine the number of living cells.

### 2.6 Collection of DPSCs-CM

After 70%–80% confluence, the growth medium of DPSCs was removed and replaced with serum-free α-MEM. After 2 days, the supernatants were collected and centrifuged at 3,000 rpm for 10 min. Then, the supernatants, which were referred to as DPSC conditioned medium (DPSCs-CM), were filtered by a 0.22-μm filter unit and stored at −80°C for further experiments.

### 2.7 Co-culture of DPSC-CM and PC12 cells *in vitro*

The PC12 cells were obtained from the American Type Culture Collection (ATCC) and cultured in advanced DMEM/F12 medium supplemented with 5% FBS and 1% penicillin/streptomycin. The PC12 cells were divided into three groups according to the culture conditions: the control group (DMEM/F12), NGF group (50 ng/mL NGF + DMEM/F12), and DPSC group (50% DPSCs-CM + DMEM/F12). The culture medium was replaced every 2 days. After 7 days, the surface marker MAP2 of PC12 cells was analyzed by immunofluorescence staining. ImageJ software was used to evaluate the average neurite length and the fluorescence intensity of MAP2.

### 2.8 Establishment of the animal model

A total of 24 female Sprague-Dawley rats, weighing 250–300 g, were purchased from the animal center of the Chinese Academy of Science (Shanghai, China). All rats were kept in specific pathogen-free (SPF) conditions at a room temperature of 22°C–24°C and 12 h light/dark cycle. All rats had water and food *ad libitum*. The experimental procedures were performed under the regulations of the Institutional Animal Care and the Ethics Committee of Wenzhou Medical University (wydw 2020–0897). All rats were randomly divided into three groups: control, GeLMA-SQ (injected with GeLMA hydrogel), and GeLMA/bFGF/DPSCs-SQ groups (injected with GeLMA/bFGF/DPSCs hydrogel). The left optic nerve was crushed intraorbitally, as previously described (Jacobi et al., 2022). After being anesthetized, a 1-cm incision extended from the temporal side of each left eye was made to expose the optic nerve. Then, the optic nerve was crushed at 1 mm for 10 s by blood vessel forceps. After the optic nerve tissue was extruded from the injured site, 5 μL of hydrogels was immediately injected into the crush site of the optic nerve using 1-mL syringes. The rats that had not undergone any kind of surgery were defined as the control group. The optic nerve was collected after 14 days for further analyses.

### 2.9 Assessment of RGC survival

RGCs survival was determined by counting the number of βIII-tubulin-positive RGCs in the retina. The retinas were carefully removed from the rats after they were transcardially infused with cold PBS and 4% paraformaldehyde (PFA). The retinas were fixed with 4% PFA for 1 h and blocked with PBS containing 5% normal goat serum for 6 h. Then, the retinal whole mounts were incubated with the primary antibody βIII-tubulin overnight at 4°C. Following PBS washes, the retinas were incubated with the secondary antibody for another 1 h at room temperature. RGCs were counted at three different retinal views.

### 2.10 Assessment of axon regeneration

In order to trace the regenerated axons, cholera toxin B fragments (CTBs) were intraocularly injected 2 days before the animals were harvested, as previously described (Cameron et al., 2020). The quantity and length of CTB-labeled regenerated axons were counted on the optic nerve slices under a fluorescence microscope. The number of regenerated axons was counted at distances (d) of 100, 300, 500, and 700 μm from the original site to the injured site. The average number of axons was calculated from the three parts of each optic nerve. The number of axons per millimeter was calculated from the measurement (2r) of the cross-sectional width of the nerve. ∑ad was the total number of axons extending for a distance in the nerve of radius r. It was estimated by summing all cross sections of thickness t (10 mm) and was calculated as follows:

$$\sum \text{ad}=\pi r^{2}\times \frac{\left[\frac{\text{average}\;\text{axons}}{\text{mm}}\right]}{t}.$$

### 2.11 Histological analysis

The histological sections were derived from the optic nerve segments that were embedded in optimal cutting temperature (OCT, Sakura, American). The cryosections (10 μm) were stained with hematoxylin and eosin (HE) and observed using a light microscope (TS100, Nikon, Japan). In addition, after euthanization, the optic nerve segments were picked out and fixed with 2.5% glutaraldehyde for 2 h, further fixed with 1% osmium tetroxide for another 2 h, dehydrated by ethanol and embedded in Epon812, cut into 50–60 nm sections, and stained with lead–uranium for transmission electron microscopy (TEM, H-600, Hitachi, Japan) observation. ImageJ was used to measure the diameter of the myelin sheath at each location.

### 2.12 Statistical analysis

All the data were presented in mean ± standard deviation (SD). The unpaired two-tailed *t*-test was used for comparisons between two groups. One-way analysis of variance (ANOVA) was used, followed by Tukey’s multiple comparison test for comparisons among three or more groups. *P* < 0.05 was considered significantly statistical. Statistical analyses were calculated by SPSS 19.0 (SPSS, Chicago, IL).

## 3 Results

### 3.1 Morphology and degradation of GeLMA-SQ and GeLMA/bFGF-SQ hydrogels

**FIGURE 1:**
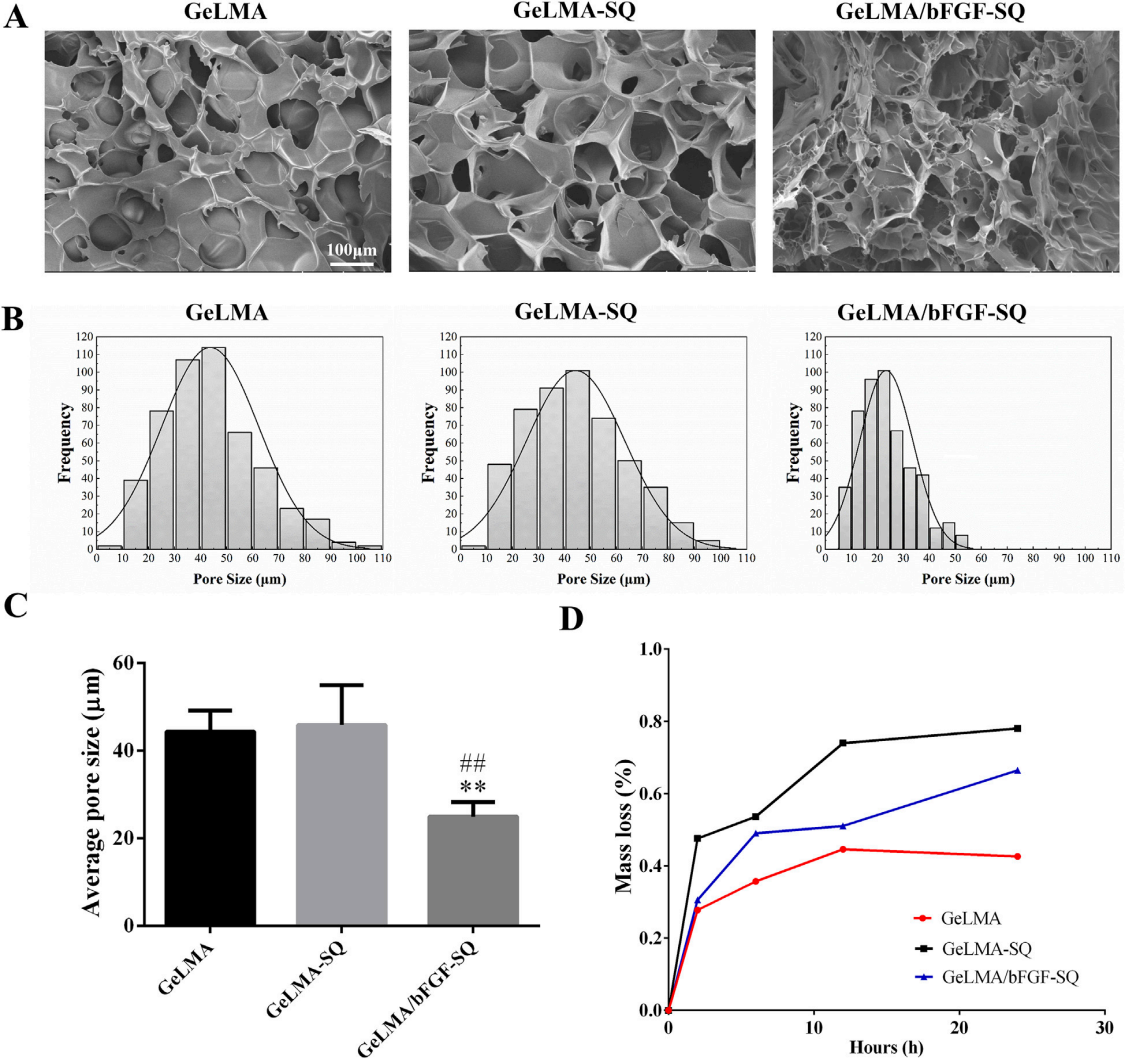
Morphology and degradability of hydrogels before and after extrusion *in vitro*. **(A)** SEM images. **(B)** Pore diameter distributions. **(C)** Average pore diameter of hydrogels. **(D)** Degradation profile of hydrogels *in vitro* (x ± SD, n = 3, ^**^ *p* < 0.01 denotes significant differences compared with the GeLMA group; ^##^ *p* < 0.01 denotes significant differences compared with the GeLMA-SQ group).

Figure 1 shows the morphology and degradation of GeLMA-SQ and GeLMA/bFGF-SQ hydrogels. As shown in Figure 1A, the results showed that the large pore size in GeLMA hydrogel was similar to that in GeLMA-SQ hydrogel, whereas the pore size in GeLMA/bFGF-SQ hydrogel was significantly decreased with the addition of bFGF. In addition, the median pore diameter of GelMA and GeLMA-SQ hydrogels was nearly 45 μm, but it reduced to 20 μm in GeLMA/bFGF-SQ hydrogel (Figure 1B). Furthermore, there was a statistical difference in the average pore size between the GeLMA and GeLMA/bFGF-SQ hydrogels, as well as the GeLMA-SQ and GeLMA/bFGF-SQ hydrogels (Figure 1C). In addition, the degradation rate was the fastest in GeLMA/bFGF-SQ hydrogel, followed by the GeLMA-SQ hydrogel, while the GeLMA hydrogel degraded slowly. Specifically, the GeLMA hydrogel displayed a reduction of nearly 40% at 24 h, while the GeLMA-SQ and GeLMA/bFGF-SQ hydrogels reached degradation levels of approximately 65% and 80%, respectively (Figure 1D).

### 3.2 Biocompatibility of GeLMA and GeLMA/bFGF hydrogels with DPSCs

**FIGURE 2:**
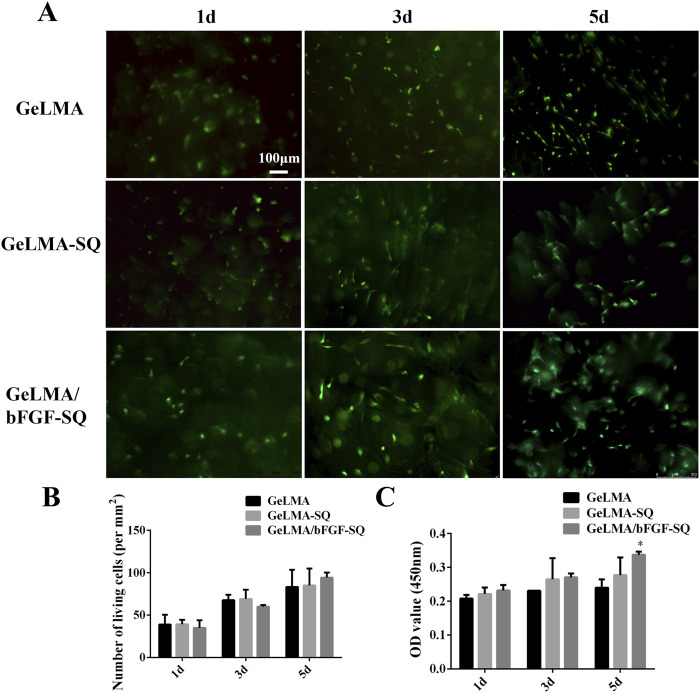
Biological compatibility of hydrogels before and after extrusion. **(A)** Live/dead staining of DPSCs in hydrogels. **(B)** Quantification of the living cells in hydrogels. **(C)** Cell proliferation of DPSCs in hydrogels at 1, 3, and 5 days (x ± SD, n = 3, ^*^ *p* < 0.05 denotes significant differences compared with the GeLMA group).

As shown in Figure 2, the results revealed that the hydrogels had great cytocompatibility with DPSCs after being co-cultured for 1, 3, and 5 days. In addition, the morphology of DPSCs gradually elongated from day 3 to day 5, especially in the GeLMA/bFGF-SQ hydrogel (Figure 2A). However, the number of living cells had no significant difference among the three hydrogels (Figure 2B). Moreover, the CCK-8 results suggested that DPSCs also had good proliferation in all the hydrogels, and the trend of data was consistent with live/dead staining (Figure 2C).

### 3.3 DPSC-CM stimulated PC12 axon growth

**FIGURE 3:**
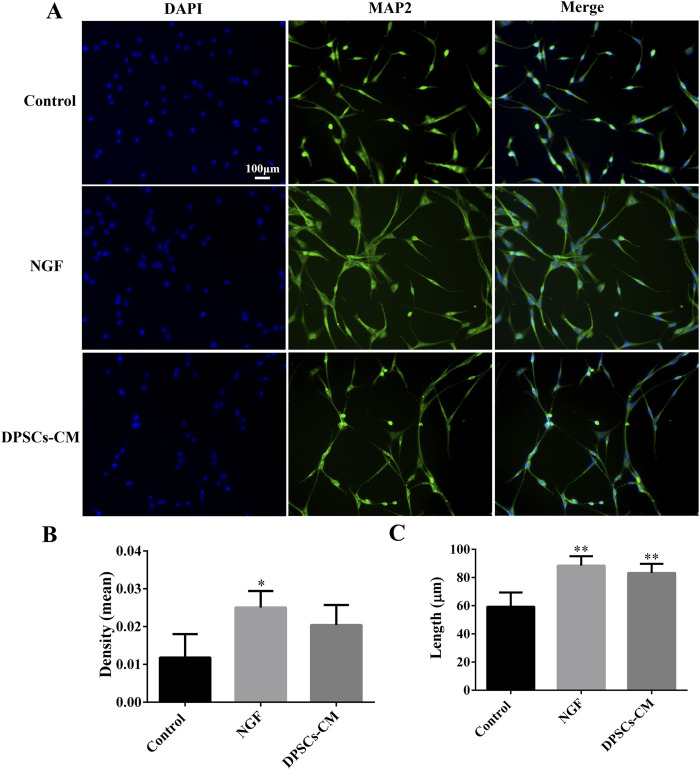
DPSCs-CM-mediated PC12 axon growth. **(A)** Immunofluorescence staining of mature neuronal marker MAP2. **(B)** Statistical analysis of MAP2 fluorescence intensity. **(C)** Statistical analysis of PC12 axon length (x ± SD, n = 3, ^*^ *p* < 0.05, ^**^ *p* < 0.01 denotes significant differences compared with the control group).

The PC12 cells were stained with MAP2 to identify the axons’ length. According to immunofluorescence staining, PC12 cells treated with NGF and DPSCs-CM had better growth of axons than in the control group (Figure 3A). Quantification of the MAP2 fluorescence intensity showed a statistically significant difference between the control and NGF groups. However, there was no statistically significant difference between the DPSC-CM and control groups, as well as the NGF group (Figure 3B). However, there was a statistically significant difference in the length of axon growth between the control and NGF groups, as well as the DPSC-CM group (Figure 3C). These results indicated that DPSCs-CM might be able to encourage the growth of the axons but not the proliferation of PC12 cells.

### 3.4 The survival of RGCs

**FIGURE 4:**
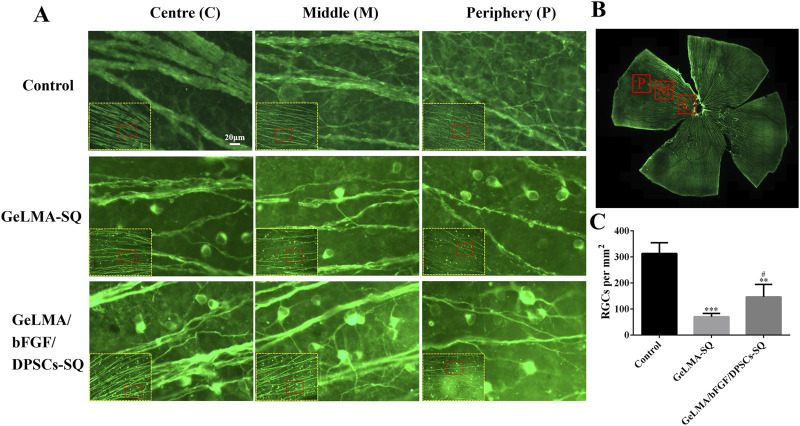
Expression of RGCs in the retina. **(A)** Images of RGCs in the retina. **(B)** Images of the clover-shaped retina after cutting. **(C)** Statistical analysis of the average number of RGCs in a single field of vision in the retina (x ± SD, n = 3, ^***^ *p* < 0.001 denotes significant differences compared with the control group; ^#^ *p* < 0.05 denotes significant differences compared with the GeLMA-SQ group).

The survival of RGCs was analyzed by counting the number of βIII-tubulin-positive labeled RGCs in the retina. From center to periphery sites, RGCs were positively labeled with βIII-tubulin, and the results suggested that the optic nerve axons were the thickest in the control group, followed by those in the GeLMA/bFGF/DPSCs-SQ group, and the axons were the thinnest in the GeLMA-SQ group (Figures 4A, B). In addition, the number of RGCs in the control group was significantly greater than that in the GeLMA-SQ and GeLMA/bFGF/DPSCs-SQ groups. Compared to the GeLMA-SQ group, the number of RGCs was much higher in the GeLMA/bFGF/DPSCs-SQ group, and the difference was statistically significant (Figure 4C).

### 3.5 Evaluation of regeneration of axons

**FIGURE 5:**
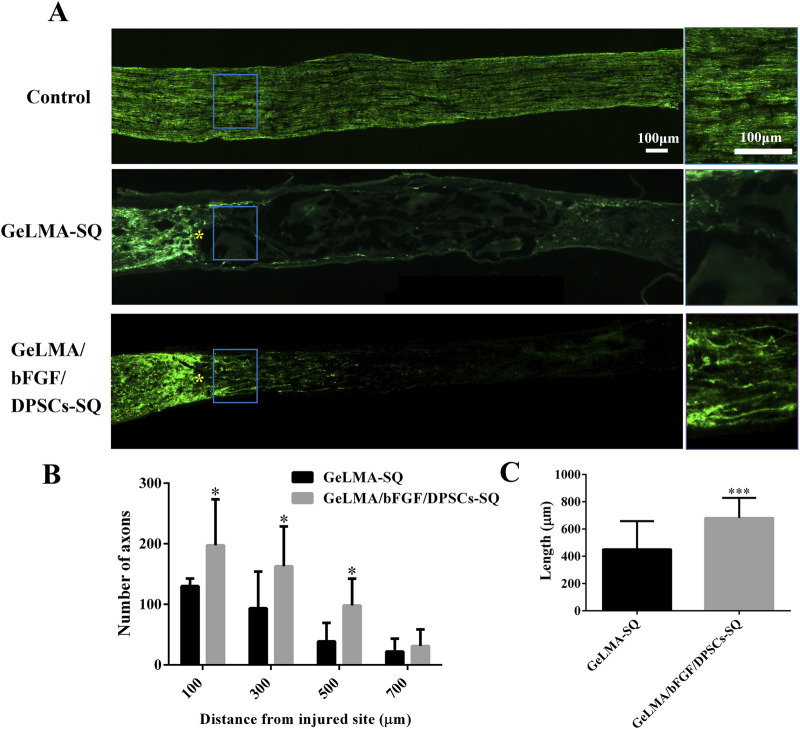
Regeneration of axons of optic nerve injury. **(A)** Regenerated axons were labeled with CTB fragment (the blue solid frame was the enlarged area at the regenerated axons, and yellow “*” was the starting point of damage). **(B)** Statistical analysis of the number of regenerated axons between the GeLMA-SQ and GeLMA/bFGF/DPSCs-SQ groups. **(C)** Statistical analysis of the length of regenerated axons between the GeLMA-SQ and GeLMA/bFGF/DPSCs-SQ groups (x ± SD, n = 3, ^*^ *p* < 0.05, ^***^ *p* < 0.001 denotes significant differences compared with the GeLMA-SQ group).

Figure 5 displays the outcomes of anterograde tracing with CTB on the optic nerve. In the GeLMA/bFGF/DPSCs-SQ group, more regenerated axons were found in the proximal crush site, but there was little regeneration of axons in the lesion site of the GeLMA-SQ group (Figure 5A). Compared with the GeLMA-SQ group, the number of CTB-positive-labeled axons was significantly increased at distances of 100, 300, and 500 µm in the GeLMA/bFGF/DPSCs-SQ group (Figure 5B). In addition, the average length of regenerated axons in the GeLMA/bFGF/DPSCs-SQ group was longer than that in the GeLMA-SQ group, with a statistically significant difference (Figure 5C).

### 3.6 HE staining and transmission electron microscope (TEM)

**FIGURE 6:**
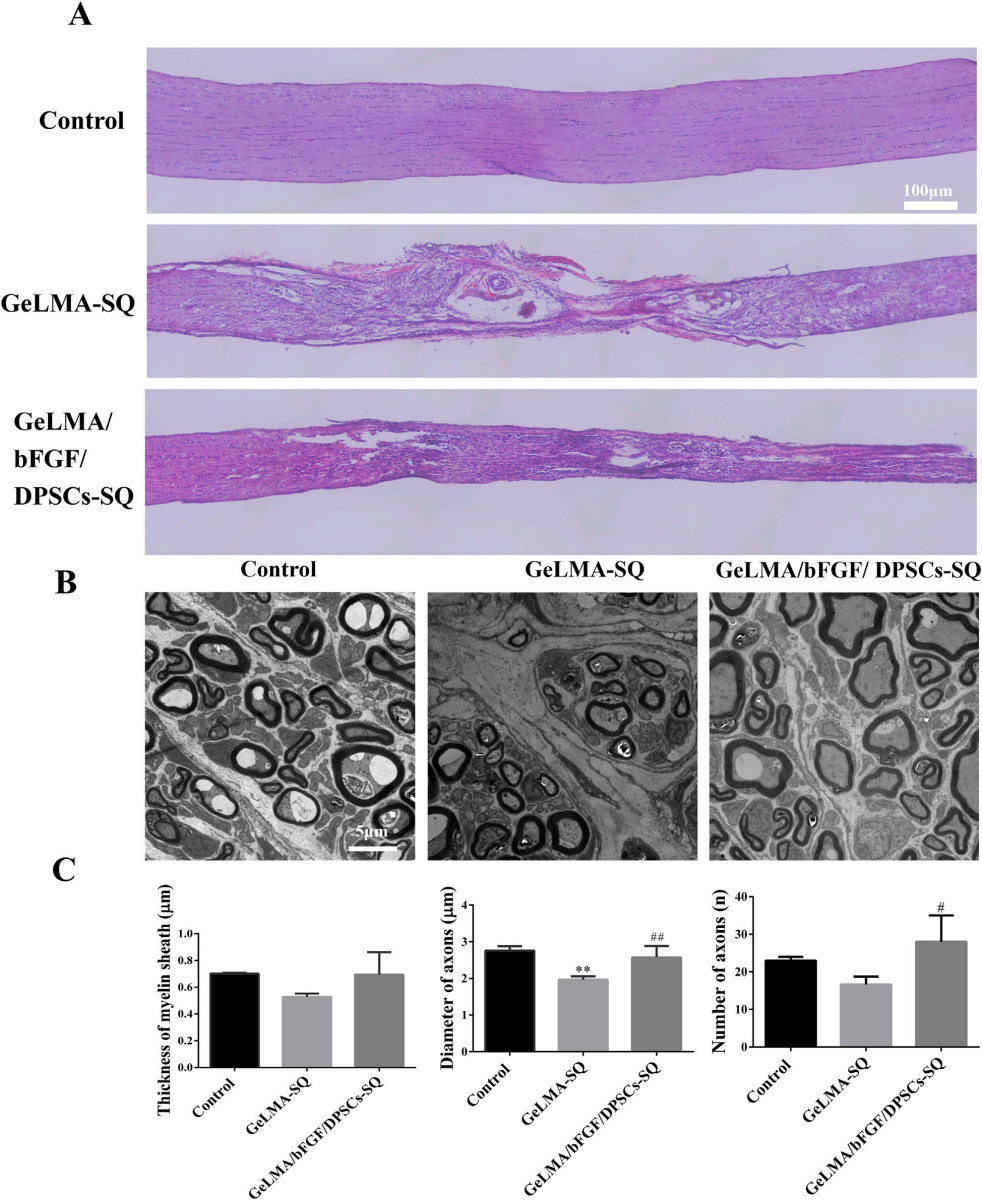
HE staining and TEM image of the optic nerve after injury. **(A)** HE images of longitudinal sections of the optic nerve. **(B)** TEM observation of optic nerve myelin sheath. **(C)** Quantification of thickness of myelin sheath and the diameter and number of axons (x ± SD, n = 3, ^**^ *p* < 0.01 denotes significant differences compared with the control group; ^#^ *p* < 0.05, ^##^ *p* < 0.01 denote significant differences compared with the GeLMA-SQ group).

As shown in Figure 6A, the results of HE staining indicated that there were obviously regenerated nerve tissue in the lesion site of the GeLMA/bFGF/DPSCs-SQ group, whereas a large number of vacuoles existed in the injured site of the GeLMA-SQ group. TEM results revealed that the optic nerve axons were arranged regularly and the myelin structure was intact in the control and GeLMA/bFGF/DPSCs-SQ groups. In contrast, the axons of the optic nerve were disorganized and several of the myelin sheaths were thin in the GeLMA-SQ group (Figure 6B). Compared to the GeLMA-SQ group, the thickness of the myelin sheath was thicker than that in the control and GeLMA/bFGF/DPSCs-SQ groups but with no significant differences. Furthermore, the diameter and number of axons in the GeLMA/bFGF/DPSCs-SQ group were higher than those in the GeLMA-SQ group, and the differences were statistically significant (Figure 6C).

## 4 Discussion

GeLMA hydrogel has garnered considerable interest in the field of tissue engineering and regenerative medicine because of its remarkable characteristics such as the 3D porous structure, great degradability, and excellent cytocompatibility, among others (Yi et al., 2024; Huang et al., 2024). In our study, even after being squeezed by the syringe, both GeLMA-SQ and GeLMA/bFGF-SQ hydrogels maintained the porous structures and exhibited great degradability (Figure 1). Some studies had demonstrated that the pore size of the scaffold had a strong effect on the series of cells’ biological behaviors, such as the adhesion, migration, survival, and proliferation properties (Buenzli et al., 2020). Meanwhile, the appropriate pore size of the scaffold required for cell infiltration and survival depended on the cell type, the diameter of most cell types ranged at 5–150 μm, and, in general, the size was 7–10 μm (Zhang et al., 2022). Our data demonstrated that the pore size of GeLMA and GeLMA-SQ hydrogels was nearly 10–100 μm, while the pore size of the GeLMA/bFGF-SQ hydrogel was 10–50 μm (Figure 1B), which was consistent with the previous description of the average pore size of GeLMA hydrogels, which was 26–103 μm (Okada et al., 2016). Compared with the GeLMA and GeLMA-SQ hydrogels, the GeLMA/bFGF-SQ hydrogel was more favorable for DPSC attachment and proliferation (Figure 2C). These results indicated that the squeezing action had no negative effect on the physical–chemical and biological properties of the GeLMA hydrogel, whereas the addition of bFGF would decrease the pore size and enhance DPSCs proliferation in the GeLMA/bFGF-SQ hydrogel, which had the similar trend in our previous research (Luo et al., 2021a).

After optic nerve injury, the lack of trophic factors will lead to RGC apoptosis, which is one of the important effects on the regeneration of the optic nerve (Junyi et al., 2015). RGCs are the specialized cells that transfer visual information to the brain, and their axons form the optic nerve; once RGCs undergo apoptosis or are dead, the vision will be impaired even if the rest of the visual system is healthy (Li et al., 2021). Recently, the transplantation of MSCs has shown great potential strategies in the treatment of traumatic optic neuropathy (Tan et al., 2022; Yang et al., 2024; Sung et al., 2020). The key characteristic of MSCs was the secretion of large neurotrophic factors to provide strong neuroprotective effects *in situ* after transplantation (Junyi et al., 2015; Li et al., 2021; Johnson et al., 2010). In our previous studies, DPSCs were one kind of adult MSCs, derived from the neural crest, which had been confirmed to have outstanding capabilities for the repair and regeneration of injured nerves in the central nervous system. Moreover, combining DPSCs with bFGF had the best effect on the treatment of damaged nerves (Albashari et al., 2020; 2024; Luo et al., 2018). Additionally, some studies demonstrated that DPSCs secreted the much higher amounts of neurotrophic genes and factors (e.g., NGF, BDNF, and PDGF) than the other MSCs types, such as BM-MSCs, which meant that DPSCs might be more effective in neuroprotection and neuroregeneration (Mead et al., 2013; Xing et al., 2023). There were studies that indicated that neurotrophin family members such as NGF and BDNF bound to tyrosine receptor kinase to trigger the classical PI3K/AKT and Ras/Raf/ERK pathways, which had the ability to impact the axon growth (Cen et al., 2023; Reichardt, 2006). In this study, the results indicated that DPSCs-CM had ability to promote the axons’ growth of PC12 cells, which was similar to NGF effects (Figure 3). In addition, the injection of GeLMA/bFGF/DPSCs-SQ hydrogel produced a prompt increase in the number of RGCs and the regeneration of axons of the optic nerve (Figures 4, 5). The data represented that the combination of DPSCs and bFGF could promote RGCs survival and the optic nerve regeneration through the paracrine effect, which was fitted for the mechanism of MSC-based therapies in traumatic optic neuropathy (Junyi et al., 2015; Li et al., 2021).

Furthermore, studies indicated that demyelination of regenerated axons was one of the critical limiting factors for the inadequate signal transduction of regenerated nerves. A lack of myelination was disadvantageous for the functional recovery of the regenerating RGC axons as they needed transfer action potentials over long distances to the retino-recipient areas (Bei et al., 2016). DPSCs had been confirmed to have the function of repairing and regenerating the myelin sheath of new nerves (Li et al., 2023). In our study, the results showed that the area of demyelination decreased and myelin sheath thickness increased in the GeLMA/bFGF/DPSCs-SQ group compared with that in the GeLMA-SQ group (Figure 6), suggesting that DPSCs in combination with bFGF had positive effects on promoting the myelination of RGC axons to further enhance the optic nerve repair.

## 5 Conclusion

In this study, our results indicated that the transplantation of 5% GeLMA hydrogel encapsulating DPSCs and bFGF via cross-linking with LED light initiation *in situ* could provide a suitable microenvironment for the repair of optic nerve injury. Among them, DPSCs in combination with bFGF protected the RGCs from apoptosis and promoted optic nerve regeneration through the paracrine mechanism via secreting a series of neurotrophic factors. Although extensive stem-cell banking currently exists around the world, its clinical application was subject to technical and regulatory issues. The paracrine mechanisms of stem cells had been increasingly explored, including the production of extracellular vesicles, cytokines, and chemokines. In the future, these small molecules might not only replace stem cells in their powerful therapeutic role but also avoid the typical problems associated with cell therapy.

## Data Availability

The original contributions presented in the study are included in the article/supplementary material; further inquiries can be directed to the corresponding authors.
